# Advancing Craniopharyngioma Management: A Systematic Review of Current Targeted Therapies and Future Perspectives

**DOI:** 10.3390/ijms25020723

**Published:** 2024-01-05

**Authors:** Edoardo Agosti, Marco Zeppieri, Sara Antonietti, Amedeo Piazza, Tamara Ius, Marco Maria Fontanella, Alessandro Fiorindi, Pier Paolo Panciani

**Affiliations:** 1 Division of Neurosurgery, Department of Medical and Surgical Specialties, Radiological Sciences and Public Health, University of Brescia, Piazza Spedali Civili 1, 25123 Brescia, Italy; edoardo_agosti@libero.it (E.A.);; 2 Department of Ophthalmology, University Hospital of Udine, p.le S. Maria della Misericordia 15, 33100 Udine, Italy; 3 Department of Neurosurgery, “Sapienza” University, 00185 Rome, Italy; 4 Neurosurgery Unit, Head-Neck and NeuroScience Department, University Hospital of Udine, p.le S. Maria della Misericordia 15, 33100 Udine, Italy

**Keywords:** craniopharyngiomas, target therapies, molecular patterns, systematic reviews, outcomes

## Abstract

Craniopharyngiomas present unique challenges in surgical management due to their proximity to critical neurovascular structures. This systematic review investigates genetic and immunological markers as potential targets for therapy in craniopharyngiomas, assessing their involvement in tumorigenesis, and their influence on prognosis and treatment strategies. The systematic review adhered to PRISMA guidelines, with a thorough literature search conducted on PubMed, Ovid MED-LINE, and Ovid EMBASE. Employing MeSH terms and Boolean operators, the search focused on craniopharyngiomas, targeted or molecular therapy, and clinical outcomes or adverse events. Inclusion criteria encompassed English language studies, clinical trials (randomized or non-randomized), and investigations into adamantinomatous or papillary craniopharyngiomas. Targeted therapies, either standalone or combined with chemotherapy and/or radiotherapy, were examined if they included clinical outcomes or adverse event analysis. Primary outcomes assessed disease response through follow-up MRI scans, categorizing responses as follows: complete response (CR), near-complete response (NCR), partial response, and stable or progressive disease based on lesion regression percentages. Secondary outcomes included treatment type and duration, as well as adverse events. A total of 891 papers were initially identified, of which 26 studies spanning from 2000 to 2023 were finally included in the review. Two tables highlighted adamantinomatous and papillary craniopharyngiomas, encompassing 7 and 19 studies, respectively. For adamantinomatous craniopharyngiomas, Interferon-2α was the predominant targeted therapy (29%), whereas dabrafenib took precedence (70%) for papillary craniopharyngiomas. Treatment durations varied, ranging from 1.7 to 28 months. Positive responses, including CR or NCR, were observed in both types of craniopharyngiomas (29% CR for adamantinomatous; 32% CR for papillary). Adverse events, such as constitutional symptoms and skin changes, were reported, emphasizing the need for vigilant monitoring and personalized management to enhance treatment tolerability. Overall, the data highlighted a diverse landscape of targeted therapies with encouraging responses and manageable adverse events, underscoring the importance of ongoing research and individualized patient care in the exploration of treatment options for craniopharyngiomas. In the realm of targeted therapies for craniopharyngiomas, tocilizumab and dabrafenib emerged as prominent choices for adamantinomatous and papillary cases, respectively. While adverse events were common, their manageable nature underscored the importance of vigilant monitoring and personalized management. Acknowledging limitations, future research should prioritize larger, well-designed clinical trials and standardized treatment protocols to enhance our understanding of the impact of targeted therapies on craniopharyngioma patients.

## 1. Introduction

Craniopharyngiomas, though histologically benign, are notorious for their challenging clinical course and the potential for significant morbidity. Traditional therapeutic approaches, including surgical resection and radiation therapy, have been associated with considerable complications and high rates of recurrence [1,2,3]. These tumors often infiltrate critical structures in the hypothalamic–pituitary axis, leading to profound endocrine and neurological consequences. The limited efficacy and substantial treatment-related complications associated with conventional therapies have underscored the need for innovative approaches to enhance therapeutic outcomes and minimize adverse effects [4].

Recent breakthroughs in molecular understanding have shed light on the genetic underpinnings of craniopharyngiomas. Notably, the identification of the BRAF-V600E mutation in a subset of these tumors has opened avenues for targeted therapeutic interventions. The emergence of BRAF inhibitors, such as vemurafenib and dabrafenib, and their combinations with MEK inhibitors, has demonstrated remarkable efficacy in select cases. This represents a paradigm shift in craniopharyngioma management, as these targeted therapies offer the potential for more precise and less invasive interventions compared with traditional approaches [1,5].

The application of targeted therapies in craniopharyngioma management holds several potential advantages. Unlike conventional treatments that often result in collateral damage to surrounding healthy tissues, targeted therapies aim to specifically inhibit the molecular pathways driving tumor growth. This precision allows for the reduction in treatment-related complications and the preservation of critical neurological and endocrine functions. Additionally, the observed responses, including substantial reductions in tumor size and improved clinical outcomes, highlight the promise of targeted therapies in achieving more favorable long-term results [6]. The ability of targeted therapies to address the underlying genetic alterations in craniopharyngiomas suggests a personalized approach to treatment, tailoring interventions based on the unique molecular profile of each patient’s tumor. Moreover, the documented responses in cases refractory to traditional therapies suggest the potential for targeted therapies to serve as salvage options, providing hope for patients with limited alternative treatment options [1,3,6].

This systematic literature review aims to provide a comprehensive synthesis of the current state of craniopharyngioma management with a specific emphasis on targeted therapies. By analyzing and synthesizing existing evidence, this review seeks to delineate the efficacy, safety profile, and long-term outcomes associated with targeted therapies in craniopharyngioma patients. Additionally, this review aims to identify existing gaps in knowledge, potential challenges, and areas warranting further research to guide future directions in the field.

## 2. Methods

### 2.1. Literature Review

This systematic review was performed following the Preferred Reporting Items for Systematic Reviews and Meta-Analysis (PRISMA) guidelines [7]. Two authors performed a systematically comprehensive literature search of the databases PubMed, Ovid MEDLINE, and Ovid EMBASE. The first literature search was performed on 1 September 2023, and the search was updated on 19 November 2023. A combination of keyword searches was performed to generate a search strategy. The search keywords, including “craniopharyngiomas”, “targeted therapy”, “outcome”, and “adverse events”, were used in both AND and OR combinations. Studies were retrieved using the following Medical Subject Heading (MeSH) terms and Boolean operators: (craniopharyngioma OR CP) AND (targeted therapy OR molecular therapy) AND (complete response OR partial response OR stable disease OR progression disease OR adverse event OR complication). Other pertinent articles were identified through reference analysis of selected papers. All studies were selected based on the following inclusion criteria: (1) English language; (2) clinical trials, including single-arm or double-arm studies, and among them randomized controlled or non-randomized controlled trials; (3) studies on adamantinomatous or papillary craniopharyngiomas, (4) studies on targeted therapies, both as a stand-alone therapy and combined therapy with CT and/or RT; and (5) studies including at least one clinical outcome or adverse event analyzed. The following exclusion criteria were employed: (1) editorials, case reports, case series, cohort studies, literature reviews, and meta-analyses; (2) studies that do not clearly define the methods and/or results; and (3) studies that do not report data on patient outcomes.

The list of identified studies was imported into Endnote X9 and duplicates were removed. Two independent researchers (E.A. and S.A.) checked the results according to the inclusion and exclusion criteria. A third reviewer (P.P.P.) resolved all disagreements. Then, eligible articles were subject to full-text screening.

### 2.2. Data Extraction

For each study, we abstracted the following information: authors, year and journal of publication, title, name and phase of the clinical trial, number of patients, diagnosis, outcomes, length of follow-up, treatment, and target.

### 2.3. Outcomes

The primary clinical outcomes were determined by assessing the disease response to targeted therapy, which was characterized as the alteration in lesion mass observed in follow-up magnetic resonance imaging (MRI) scans, conducted at least three months post-treatment cessation. Specifically, the response to targeted therapy was categorized as follows: complete response (CR) for disease regression exceeding 95%; near-complete response (NCR) for disease regression between 85–95%; partial response (PR) for disease regression below 85%; stable disease (SD) denoting no discernible change in lesion size based on control MRI; and progression disease (PD) indicating an increase in disease size during follow-up. Secondary outcomes were type and duration of treatments and adverse events.

### 2.4. Risk of Bias Assessment

**Figure 1:**
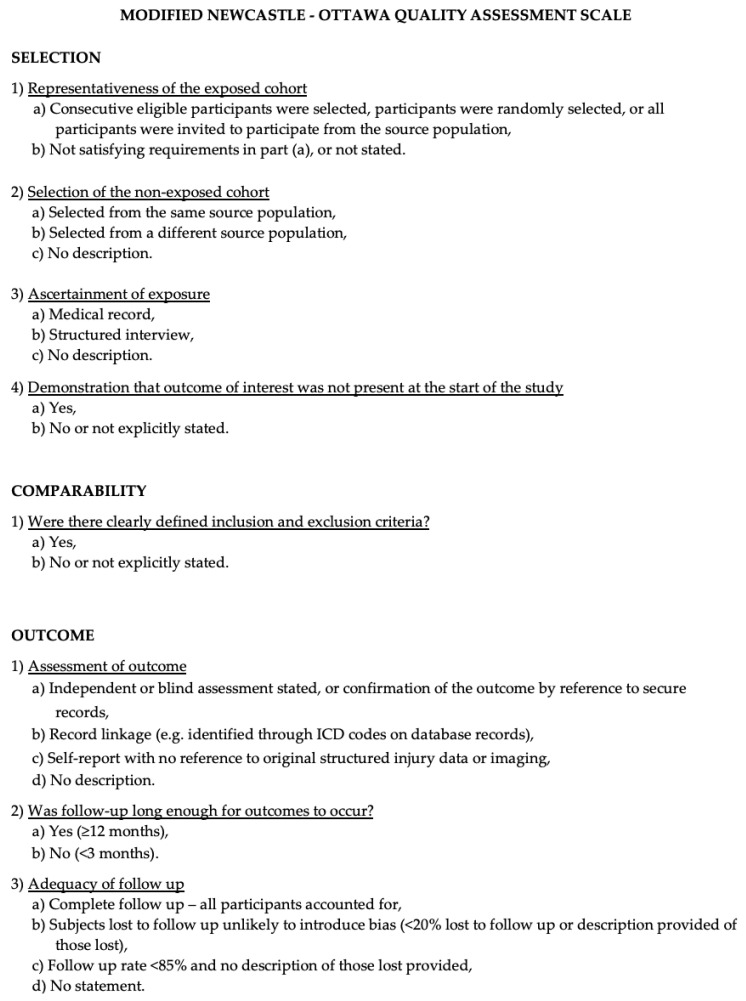
The Modified NOS.

The Newcastle–Ottawa Scale (NOS) [8] was used to assess the quality of the included studies. Quality assessment was performed by assessing the selection criteria, comparability of the study, and outcome assessment. The ideal score was 9. Higher scores indicated better quality of studies. Studies receiving 7 or more points were considered high-quality studies. Two authors (E.A. and P.P.P.) performed the quality assessment independently. When discrepancies arose, the papers were re-examined by the third author (Figure 1).

### 2.5. Statistical Analysis

Descriptive statistics were reported, including ranges and percentages. All statistical analyses were performed using the R statistical package v3.4.1 http://www.r-project.org (accessed on 10 December 2023).

## 3. Results and Discussion

### 3.1. Literature Review

**Figure 2:**
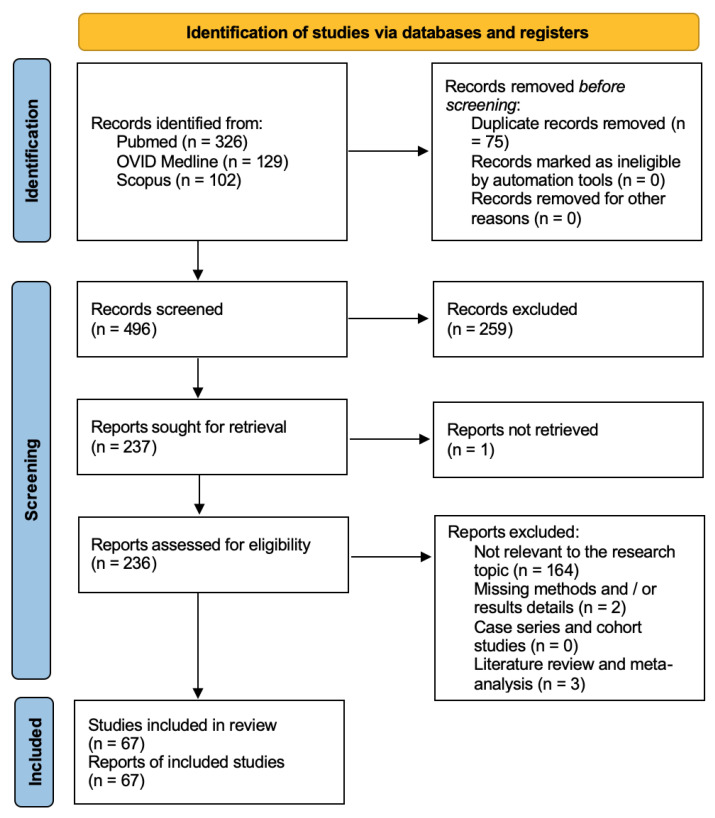
PRISMA flow chart.

A total of 891 papers were identified after duplicate removal. After title and abstract analysis, 388 articles were identified for full-text analysis. Eligibility was assessed for 386 articles and ascertained for 26 articles. The remaining 360 articles were excluded for the following reasons: (1) not relevant to the research topic (345 articles); (2) articles non-reporting selected outcomes (nine articles); (3) systematic literature review or meta-analysis (five articles); and (4) lack of method and/or results details (one article). All studies included in the analysis had at least one or more outcome measures available for one or more of the patient groups analyzed. Figure 2 shows the flow chart according to the PRISMA statement.

**Figure A1:**
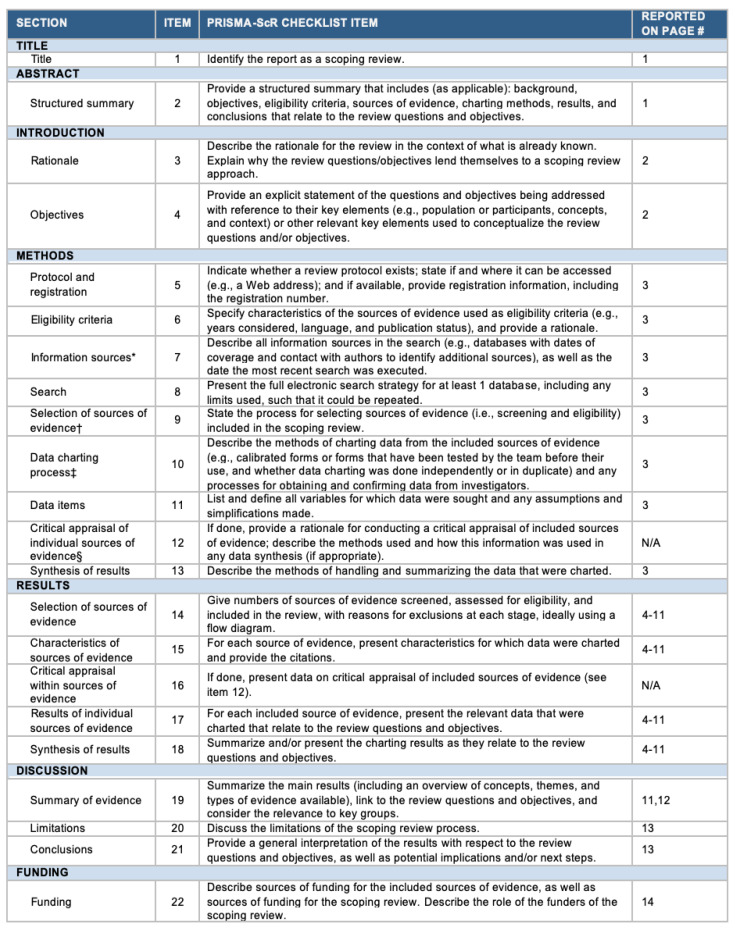
The PRISMA-ScR checklist. Abbreviation: JBI = Joanna Briggs Institute; PRISMA-ScR = Preferred Reporting Items for Systematic reviews and Meta-Analyses extension for Scoping Reviews. * Where sources of evidence (see second footnote) are compiled from, such as bibliographic databases, social media platforms, and Web sites. † A more inclusive/heterogeneous term used to account for the different types of evidence or data sources (e.g., quantitative and/or qualitative research, expert opinion, and policy documents) that may be eligible in a scoping review as opposed to only studies. This is not to be confused with information sources (see first footnote). ‡ The frameworks by Arksey and O’Malley (6) and Levac and colleagues (7) and the JBI guidance (4, 5) refer to the process of data extraction in a scoping review as data charting. § The process of systematically examining research evidence to assess its validity, results, and relevance before using it to inform a decision. This term is used for items 12 and 19 instead of “risk of bias” (which is more applicable to systematic reviews of interventions) to include and acknowledge the various sources of evidence that may be used in a scoping review (e.g., quantitative and/or qualitative research, expert opinion, and policy document).

The PRISMA Extension for Scoping Reviews (PRISMA-ScR) checklist is available in the Appendix A (Figure A1).

### 3.2. Data Analysis

A summary of the included studies reporting on targeted therapies for adamantinomatous and papillary craniopharyngiomas is presented in Table 1 and Table 2, respectively.

This systematic literature review encompasses 26 studies focusing on targeted therapies for craniopharyngiomas. Table 1 specifically addresses adamantinomatous craniopharyngiomas, featuring seven studies, while Table 2 concentrates on papillary craniopharyngiomas, involving 19 studies. The studies span from 2000 to 2023, indicating a progressive interest in exploring targeted therapies for craniopharyngiomas.

Concerning adamantinomatous craniopharyngiomas, prior treatments included subtotal resection (STR), cyst drainage, ventriculoperitoneal shunt (VPS), radiotherapy (RT), gross total resection (GTR), intracystic bleomycin, and phosphorus-32 therapy. For papillary craniopharyngiomas, prior treatments encompassed surgery with various approaches (endoscopic endonasal approach (EEA), microscopical transcranial approach (MTA)), radiotherapy (RT), and decompressive surgery.

Regarding the type and duration of targeted therapies, for adamantinomatous craniopharyngiomas, the main agents used were Interferon-2α (IFN-2α), followed by pegylated interferon-α-2b (IFN-α-2b), tocilizumab (TCZ), binimetinib, and vemurafenib. The duration range of the treatment was 3–28 months. For papillary craniopharyngiomas, the main agents used were vemurafenib, dabrafenib, and trametinib, with a treatment duration range of 1.7–28 months. Notably, in adamantinomatous craniopharyngiomas, CR was reported in four studies, PR in only one study, SD in three studies, and PD in four studies. As for papillary craniopharyngiomas, CR was reported in 13 studies, NCR and PR in one study each, and SD in nine studies.

Adverse events related to targeted therapies in adamantinomatous craniopharyngiomas included constitutional symptoms, fever, nausea, vomiting, skin changes, and various neurological symptoms. Notable events include grade 2 transaminase elevation and mild thrombocytopenia. As for papillary craniopharyngiomas, adverse events included arthralgia, low-grade fever, photosensitivity, drug-induced pyrexia, and skin-related issues.

The data analysis revealed a diverse landscape in terms of the types and durations of targeted therapies used for adamantinomatous and papillary craniopharyngiomas. Tocilizumab was the most-used targeted therapy for adamantinomatous (29%), while dabrafenib (70%) was the most-used therapy for papillary craniopharyngiomas. Encouragingly, both types of craniopharyngiomas exhibit positive responses, with a significant percentage achieving CR (29% and 32% for adamantinomatous and papillary craniopharyngiomas, respectively) or NCR (26% for papillary craniopharyngiomas). Adverse events, though common, are manageable and, in some cases, resolution is observed with treatment adjustments. This underscores the importance of vigilant monitoring and personalized management to enhance treatment tolerability.

This systematic literature review examined 26 studies on targeted therapies for craniopharyngiomas, categorizing them into adamantinomatous and papillary types. Notably, the interest in exploring targeted therapies for craniopharyngiomas has progressively grown from 2000 to 2023. Previous treatments for adamantinomatous craniopharyngiomas included various surgical and radiological approaches, while papillary craniopharyngiomas were treated with surgery and radiotherapy. Targeted therapies for adamantinomatous cases primarily involved IFN-2α, TCZ, and others, with treatment durations ranging from 3 to 28 months. Papillary cases mainly used vemurafenib, dabrafenib, and trametinib, with treatment durations ranging from 1.7 to 28 months. Positive responses, including CR and NCR, were observed in both types, with tocilizumab and dabrafenib being the most utilized. Adverse events, though common, were manageable, emphasizing the need for careful monitoring and personalized management to improve treatment tolerability.

#### 3.2.1. State of the Art of Adamantinomatous Craniopharyngioma-Targeted Therapies

The management of adamantinomatous craniopharyngiomas remains challenging, with limited consensus on optimal treatment strategies. The studies included in this review explored various targeted therapies, including IFN-α [18,24,26,31], pegylated IFN-α-2b, TCZ, bevacizumab, binimetinib, and a combination of dabrafenib and trametinib. Interferon-α has been investigated in several studies, but the outcomes have been mixed, with some cases showing disease progression. Pegylated IFN-α-2b has also been explored, with reports of both CR and PR, highlighting its potential in certain cases. Tocilizumab and bevacizumab have demonstrated efficacy in reducing cystic disease, while binimetinib showed stability. The combination of dabrafenib and trametinib yielded promising results, including complete responses with significant tumor reduction [5,14,17].

The studies on adamantinomatous craniopharyngiomas reveal a diverse range of targeted therapeutic interventions, reflecting the complexity of managing these tumors. Jakacki et al. [9] investigated the use of IFN-2α in patients with adamantinomatous craniopharyngiomas, with outcomes showing PD in several cases. Yeung et al. [10] explored the efficacy of pegylated IFN-α-2b, reporting a CR in one patient and PR in others. Grob et al. [11] employed TCZ and bevacizumab, achieving a decrease in cystic disease after re-initiation of combination therapy.

Goldman et al. [12] investigated the use of pegylated IFN-α-2b, reporting limited efficacy in both stratum 1 and stratum 2 patients. Patel et al. [13] explored binimetinib, observing SD. Vos-Kerkhof et al. [14] reported on the use of TCZ, demonstrating SD to date. De Rosa et al. [34] employed bevacizumab, achieving a PR with a 66.1% shrinkage of tumor volume at 3 months. Finally, the study by Nussbaum et al. [29] utilized dabrafenib and trametinib, showing a CR with over 95% tumor reduction after 21 months of treatment.

The variety in targeted therapies highlights the ongoing efforts to identify effective interventions for adamantinomatous craniopharyngiomas [26,33]. However, the outcomes vary, with some studies demonstrating disease control and others showing limited efficacy. It is essential to consider the heterogeneity of adamantinomatous craniopharyngiomas and the need for personalized treatment strategies. Moreover, the variability in treatment responses underscores the need for personalized and targeted approaches to adamantinomatous craniopharyngiomas [13,17]. Given the limited number of studies and the heterogeneity of patient populations, further research is needed to establish the optimal sequencing and combination of targeted therapies for this subtype.

#### 3.2.2. State of the Art of Papillary Craniopharyngioma-Targeted Therapies

Papillary craniopharyngiomas have garnered increased attention due to their distinct molecular characteristics, particularly the presence of BRAF mutations [25,29,33]. The studies included in this review focused on targeted therapies such as vemurafenib, dabrafenib, and trametinib, either as monotherapies or in combination. Vemurafenib, a BRAF inhibitor, showed limited efficacy in one study, with disease progression reported. Dabrafenib and trametinib, either as monotherapies or in combination, demonstrated more promising results. Several studies reported CR, NCR, PR, or SD in patients with papillary craniopharyngiomas [25,26,29,35].

Aylwin et al. [17] investigated vemurafenib, reporting PD in one patient after 7 months of treatment. Brastianos et al. [31] explored the combination of dabrafenib and trametinib, observing SD after 18 months. Roque et al. [18] used dabrafenib and trametinib, achieving NCR with a near disappearance of the tumor on MRI 7 months after treatment.

Several studies, including those by Rao et al. [22], Bernstein et al. [23], Di Stefano et al. [24], Khaddour et al. [25], Gopal et al. [26], Butt et al. [27], and Chik et al. [28] employed dabrafenib and trametinib combinations, reporting various responses such as CR, NCR, PR, or SD at [29] different time points. These findings underscore the potential of this combination in managing papillary craniopharyngiomas.

Other studies, such as those by Himes et al. [20], Juratli et al. [21], and Wu et al. [33], utilized dabrafenib as a monotherapy or in combination, demonstrating SD and PR. These studies contribute to the growing body of evidence on the effectiveness of BRAF inhibitors in the treatment of papillary craniopharyngiomas.

#### 3.2.3. Overall Survival and Adverse Events

Assessing overall survival (OS) and adverse events is crucial in evaluating the long-term efficacy and safety of targeted therapies for craniopharyngiomas. The studies included in this review varied in their reporting of OS and adverse events, making it challenging to draw comprehensive conclusions. However, some key observations can be made [17,30,31].

In the study by Jakacki et al. [9], which investigated IFN-2α for adamantinomatous craniopharyngiomas, the median OS was not reached during the study period. Yeung et al. [10] reported a median OS of 7.2 years in patients treated with pegylated IFN-α-2b.

For papillary craniopharyngiomas, Brastianos et al. [16] reported an estimated median progression-free survival (PFS) of 15.5 months and a median OS of 21.4 months in patients treated with dabrafenib and trametinib. Roque et al. [18] observed an estimated 18-month PFS in patients receiving the same combination.

It is important to note that these findings should be interpreted cautiously due to the heterogeneity of patient populations, treatment regimens, and study designs. Additionally, long-term follow-up data are limited, and ongoing research is needed to assess the durability of treatment responses and OS [16,17].

As for adverse events, the studies reported varying degrees of treatment-related side effects. Common adverse events associated with targeted therapies for craniopharyngiomas included fatigue, fever, nausea, vomiting, headache, and laboratory abnormalities. The severity and frequency of adverse events varied among patients, highlighting the importance of individualized treatment approaches and close monitoring [12,19,31,34].

#### 3.2.4. The Emerging Role of Stereotactic Radiosurgery Radioenhancers for the Management of Craniopharyngiomas

Craniopharyngiomas over time have presented unique therapeutic dilemmas due to their proximity to critical structures. In recent years, the exploration of advanced treatment modalities such as stereotactic radiosurgery (SRS) and radioenhancers has shown promise in transforming the management landscape for these lesions. However, despite intriguing insights provided by seminal reviews on the subject, there remains a significant knowledge gap regarding the practical implementation and effectiveness of these strategies in large-scale clinical settings.

Two comprehensive reviews, Refs. [35,36], published some time ago, shed light on the potential benefits of SRS and radioenhancers in the treatment of craniopharyngiomas. The rationale behind these innovative approaches stems from the imperative need to shield white matter and, more specifically, cranial nerves during radiosurgery protocols for these intricate skull base lesions. The reviews, authored by reputable experts in neuro-oncology, meticulously outline the theoretical foundations and early evidence supporting the use of SRS and radioenhancers in craniopharyngioma management. The first review, authored by Ganau et al. [35], delves into the historical evolution of SRS and its application in treating craniopharyngiomas. The authors emphasize the precise targeting capabilities of SRS, allowing for optimal tumor control while minimizing damage to surrounding healthy tissues. Highlighting key studies and case series, the review outlines the favorable outcomes observed in terms of tumor response and patient prognosis. Despite the compelling evidence presented, the authors acknowledge the need for more extensive, multicenter studies to establish the broader efficacy and safety profile of SRS for craniopharyngiomas. A similar sentiment echoes in the second review by Ganau et al. [36], which focuses on the integration of radioenhancers in the management of craniopharyngiomas. The review explores the potential of radioenhancers in enhancing the therapeutic ratio of radiation treatment by selectively sensitizing tumor cells. By providing an in-depth analysis of preclinical studies and early-phase clinical trials, the authors emphasize the promise of radioenhancers in increasing the effectiveness of radiation therapy for craniopharyngiomas. Yet they caution that further research is essential to validate these findings and address concerns related to long-term outcomes and potential side effects.

While these reviews lay a solid foundation for the potential roles of SRS and radioenhancers in craniopharyngioma management, a noteworthy gap exists between theory and widespread clinical adoption. Despite the detailed exploration of these strategies and their theoretical advantages, the systematic implementation of SRS and radioenhancers in large international series treating craniopharyngiomas has not yet materialized. This presents a critical juncture in neuro-oncology research, emphasizing the need for comprehensive, long-term studies that assess the real-world applicability and outcomes of these advanced therapeutic approaches.

The reluctance to adopt SRS and radioenhancers on a broader scale may stem from a variety of factors, including concerns about long-term toxicity, lack of standardized protocols, and the inherent challenges of conducting multicenter trials for rare tumor entities. Additionally, the potential benefits of these strategies may be overshadowed by the established paradigms of craniopharyngioma management, such as surgery and conventional radiotherapy, which have long been the mainstays of treatment.

Addressing this gap in knowledge and translating the theoretical advantages into clinical practice requires collaborative efforts across institutions and international neuro-oncology communities. Multicenter studies with long-term follow-ups, standardized protocols, and comprehensive data collection are imperative to ascertain the true impact of SRS and radioenhancers on craniopharyngioma outcomes. Furthermore, the incorporation of advanced imaging techniques and molecular profiling could contribute to a more nuanced understanding of treatment responses and potential predictors of success.

#### 3.2.5. The Role of Targeted Therapies for Other Skull Base Lesions

Skull base lesions encompass a diverse range of tumors, each presenting its own set of challenges in terms of location, proximity to critical structures, and treatment response. Traditional therapeutic approaches, such as surgery and radiation, have limitations, necessitating the exploration of novel treatment strategies. Targeted therapies, designed to specifically address the molecular alterations driving tumor growth, have shown promise in improving outcomes and reducing adverse effects associated with conventional treatments. Over the years, targeted therapies have emerged as promising interventions for several skull base lesions, offering a more nuanced and effective approach compared with traditional treatment modalities. In this context, of particular interest is the development of targeted therapies for skull base chordomas.

Chordomas, rare tumors derived from notochordal remnants, predominantly affect the skull base. These lesions are characterized by slow growth, local invasiveness, and a propensity for recurrence. Historically, the management of chordomas has been challenging, often requiring extensive surgical interventions. However, recent advances in molecular understanding have paved the way for targeted therapeutic approaches. A notable subset of chordomas harbors mutations in the BRAF gene, specifically the V600E mutation [16,33]. This finding has prompted investigations into the efficacy of BRAF inhibitors as a targeted therapy. Encouragingly, case reports and small-scale studies have demonstrated significant clinical and radiographic responses in patients with BRAF-mutated skull base chordomas treated with BRAF inhibitors [27]. This shift toward precision medicine marks a paradigmatic advancement in the management of chordomas, moving beyond the constraints of traditional therapeutic modalities.

BRAF inhibitors, such as vemurafenib and dabrafenib, have shown efficacy in inhibiting the aberrant signaling associated with BRAF mutations. In chordomas, where the BRAF V600E mutation is prevalent, these inhibitors have demonstrated promising results. A case series reported dramatic clinical and radiographic responses in patients treated with BRAF/MEK inhibitors, highlighting the potential of this targeted approach [27]. While BRAF inhibitors target the molecular drivers of chordomas, immunomodulatory therapies are emerging as additional strategies. Tocilizumab, which targets IL-6, has shown promise in craniopharyngiomas [11]. Given the intricate immune landscape of chordomas, exploring immunotherapeutic agents may unveil new dimensions in treatment.

Despite the optimism surrounding targeted therapies for skull base chordomas, challenges persist. The rarity of these tumors poses difficulties in conducting large-scale clinical trials, limiting the generalizability of findings. Concerns about long-term toxicity, the need for standardized protocols, and a deeper understanding of treatment responses underscore the ongoing complexities in translating targeted therapies into routine clinical practice [29,31]. Addressing these challenges may involve exploring combination therapies. The synergy between targeted agents and traditional treatments, such as surgery or radiation, could enhance therapeutic efficacy. Moreover, investigating the tumor microenvironment and identifying potential vulnerabilities beyond specific mutations may guide the development of combination strategies [35].

#### 3.2.6. Limitations and Future Directions

Although this systematic literature review contributes valuable insights, it is essential to recognize and address several limitations inherent in the included studies. The considerable heterogeneity across these studies, encompassing differences in study design, patient demographics, treatment modalities, and outcome assessment, poses a substantial challenge in conducting a meaningful meta-analysis or deriving definitive conclusions. Furthermore, the restricted number of studies and, in certain instances, the relatively modest sample sizes emphasize the imperative for undertaking more extensive, meticulously designed clinical trials [2,30]. These larger-scale trials are crucial for establishing robust evidence, enhancing the generalizability of findings, and providing a more comprehensive understanding of the nuances inherent in the subject matter.

Future research in the field of craniopharyngioma-targeted therapies should focus on prospective, multicenter studies with standardized treatment protocols and long-term follow-up. Collaboration among researchers, clinicians, and pharmaceutical companies is essential to address the rarity and heterogeneity of craniopharyngiomas and facilitate the development of targeted therapies. Biomarker-driven approaches, including molecular profiling and genetic testing, may help identify subgroups of patients who are more likely to benefit from specific targeted interventions [28,30,32,33,35].

Moreover, the integration of novel imaging techniques, such as advanced MRI and PET imaging, can contribute to accurate disease monitoring and response assessment. Long-term outcomes, including OS and quality of life, should be prioritized in future studies to provide a comprehensive understanding of the impact of targeted therapies on craniopharyngioma patients [16].

## 4. Conclusions

The examination of targeted therapies for adamantinomatous and papillary craniopharyngiomas has unveiled a varied landscape, highlighting tocilizumab as the predominant choice for adamantinomatous (29%) and dabrafenib for papillary cases (70%). The positive responses observed, with substantial rates of complete response (CR) in both types, offer optimism for the effectiveness of these therapies. Despite common adverse events, their manageable nature underscores the importance of meticulous monitoring and personalized management strategies.

While these findings contribute valuable insights, it is essential to acknowledge certain limitations in the existing literature. To address these gaps, future research endeavors should prioritize the initiation of larger, well-designed clinical trials. Standardized treatment protocols and collaborative efforts across research institutions are crucial to gaining a more profound understanding of the impact of targeted therapies on patients with craniopharyngiomas. This call for further investigation aims to refine treatment approaches, optimize patient outcomes, and ultimately advance the field of craniopharyngioma-targeted therapies.

## Figures and Tables

**Table 1:** Summary of the studies included in the systematic literature review reporting on targeted therapies for adamantinomatous craniopharyngiomas.

Author, Year	Age (Years), Sex	Prior Treatment	Systemic Targeted Treatment		Next Treatment	Outcomes	Adverse Events
			Agents	Duration (Months)			
Jakacki et al. [9] 2000	6.1, F	STR, cyst drainage, VPS	IFN-2α: induction phase: 8,000,000 U/m2 QD via SC injection for 16 weeks; maintenance phase: 8,000,000 U/m2 three times per week for an additional 32 weeks in patients without progressive disease at 16 weeks; Tylenol and/or a nonsteroidal anti-inflammatory agent were given 30 min before each INF dose during the 1st week.	12	RT	PD (25 months after initiation).	All patients had fever during the first few treatments, usually accompanied by chills and myalgias. Two patients with panhypopituitarism developed hypotension and lethargy with fever after the first or second dose of INF but recovered rapidly after receiving stress doses of steroid medications. One patient had refractory frontal lobe seizures shortly after starting treatment. One patient had hyperpigmentation; 11 lost weight during the induction phase. Most patients began to gain weight during the maintenance phase, although 3 continued to lose weight. All patients gained weight since discontinuing INF therapy. Mild nausea; constitutional symptoms lasting 1–2 days after each dose, which lessened over time. Transient constitutional symptoms after each dose, which improved over time; grade 2 transaminase elevation and mild thrombocytopenia. Nausea and occasional vomiting for 24 h after each injection; symptoms disappeared as treatment continued.
Jakacki et al. [9] 2000	10.2, F	STR, cyst drainage		7	N/A	PD (7 months after initiation).	
Jakacki et al. [9] 2000	13.4, M	STR × 2, cyst drainage, RT, GTR		12	N/A	PD (37 months after initiation).	
Jakacki et al. [9] 2000	19.2, M	STR, cyst drainage, RT		N/A	N/A	N/A	
Jakacki et al. [9] 2000	11.4, M	STR		12	N/A	PD (22 months after initiation).	
Jakacki et al. [9] 2000	7.1, M	STR		N/A	N/A	N/A	
Jakacki et al. [9] 2000	4.2, F	STR, intracystic bleomycin		12	N/A	PD (21 months after initiation).	
Jakacki et al. [9] 2000	4.2, M	STR, cyst drainage		9	N/A	PD (21 months after initiation).	
Jakacki et al. [9] 2000	13.1, M	GTR, cyst drainage, STR × 2, RT		12	N/A	SD >29 months after initiation.	
Jakacki et al. [9] 2000	11.6, M	GTR		12	N/A	SD (25 months after initiation).	
Jakacki et al. [9] 2000	13.9, M	STR, RT		12	N/A	PR >30 months after initiation.	
Jakacki et al. [9] 2000	13.3, M	GTR		12	N/A	CR > 22 months after initiation.	
Jakacki et al. [9] 2000	11.9, M	GTR × 2, phosphorus-32 × 2		12	N/A	PD 21 months after initiation.	
Jakacki et al. [9] 2000	14.5, M	GTR × 2, STR, RT, cyst drainage		N/A	N/A	N/A	
Jakacki et al. [9] 2000	19.8, F	STR × 4, phosphorus-32, RT, cyst drainage, bleomycin		3	N/A	PD 3 months after initiation.	
Yeung et al. [10] 2012	9, F	GTR	Pegylated IFN-α-2b: initial dose 3 μg/kg as weekly SC injection.	24	N/A	CR; disease-free 120 months after initiation.	Mild nausea; constitutional symptoms lasting 1–2 days after each dose, which lessened over time.
Yeung et al. [10] 2012	14, M	STR, RT (54 Gy)	Pegylated IFN-α-2b: initial dose 3 μg/kg as weekly SC injection; reduced to 1.5 mg/kg/week after 6 months.	24	N/A	PR 60 months after initiation.	Transient constitutional symptoms after each dose, which improved over time; grade 2 transaminase elevation and mild thrombocytopenia.
Yeung et al. [10] 2012	13, M	GTR × 3	Pegylated IFN-α-2b: initial dose 1 μg/kg as weekly SC injection.	15	N/A	CR 4 months after stopping treatment.	Nausea and occasional vomiting for 24 h after each injection; symptoms disappeared as treatment continued.
Yeung et al. [10] 2012	14, M	STR × 2	Pegylated IFN-α-2b: initial dose 3 μg/kg as weekly SC injection.	12	phosphorus-32.	A 30% decrease in the cystic component after 4 months of treatment; at 12 months the cyst increased in size and the patient was taken off the study and referred for treatment with radioactive phosphorus.	Transient fatigue and fevers for 2 days after each dose, which improved over time.
Yeung et al. [10] 2012	15, F	GTR	Pegylated IFN-α-2b: initial dose 1 μg/kg as weekly SC injection.	6 (ongoing to achieve 24 months).	N/A	SD (6 months after initiation).	N/A
Grob et al. [11] 2019	3, M	Intra-cystic IFN-α and bleomycin	TCZ: 12 mg/kg IV every 2 weeks; 9 months later, IV bevacizumab every 2 weeks was co-administered.	28 (14 on combination therapy).	Six months off therapy, an MRI demonstrated an overall increase in cystic mass, so combination therapy was re-initiated; levetiracetam was administered for seizures.	Decrease in cystic disease 4 months after re-initiation of combination therapy.	Neutropenia (CTCAE v5, grade 3).
Grob et al. [11] 2019	7, M	Cyst aspirations, STR (MTA), RT (54 Gy), intra-cystic bleomycin.	TCZ: 12 mg/kg IV every 2 weeks.	7	N/A	PR (after 6 months of therapy, from 26 × 28 × 27 mm to 18 × 24 × 18 mm).	N/A
Goldman et al. [12] 2020	2–25 (18 Pts), M (8 Pts) F (10 Pts)	Stratum 1: patients treated with surgery alone and who had never received radiotherapy; stratum 2: patients who had previously received radiotherapy.	Weekly SC injection of pegylated interferon alpha-2b (either PEG-Intron or Sylatron, based on availability) at a dose of 1 µg/kg/week; 6 weekly doses constituted a course; treatment could continue without break for up to 18 courses.	24	N/A	Stratum 1.—only 1 patient met the protocol definition of PR; stratum 1 was closed prematurely. Stratum 2—None of the 11 patients attained the primary endpoint of objective radiographic response, and the study was closed.	The most frequently reported toxicities were: decreased white blood cells and neutrophils, elevated alanine aminotransferase (ALT)/aspartate aminotransferase, and fever. There were 12 grade 3 toxicities over the 141 cycles given. Two patients experienced a total of 4 episodes of neutropenia and one patient experienced 2 episodes of grade 3 increase in ALT. Two of the eighteen patients came off therapy due to toxicity: 1 patient (stratum 2) refused further study treatment secondary to grade 2 flu-like symptoms after receiving only a single dose of peginterferon, and another patient (stratum 1) came off treatment after 3 cycles due to grade 3 ALT elevation. One additional patient in stratum 2 was dose-reduced during cycle 5 secondary to grade 3 anorexia.
Patel et al. [13] 2021	26, F	VPS Surgery x8, CT, RT, GKRS.	Binimetinib: 45 mg BID → 30 mg BID → 30 mg in the morning and 15 mg in the evening due to adverse effects.	8	N/A	SD (since December 2019).	Furuncles/papulopustular rash on tights and buttocks, nail dystrophy, hyponatremia, venous stasis with poor wound healing, fatigue/daytime sleepiness, and weight gain.
Vos-Kerkhof et al. [14] 2023	15, F	EVD; Surgery: STR; STR (EEA × 1; MTA × 1) VPS STR (combined left transcranial and transsphenoidal approach).	TCZ (20 mg/mL) 800 mg every 2 weeks IV during 1 h.	9	N/A	SD (To date, from the start of TCZ, both the residual cystic and solid components of the craniopharyngioma have remained stable).	N/A
De Rosa et al. [15] 2023	84, M	STR, neuroendoscopic cyst fenestration.	Bevacizumab (10 mg/kg) every 2 weeks.	3	VMAT RT	PR (66.1% shrinkage: of tumor volume at 3 months).	N/A

Abbreviations: BID = twice daily; CT = chemotherapy; CR = complete response; CTCAE = common terminology criteria for adverse events; EEA = endoscopic endonasal approach; EVD = external ventricular drainage; GKRS = gamma knife radiosurgery; GTR = gross total resection; INF = interferon; IV = intravenous; MTA = microscopical transcranial approach; N/A = not applicable; PD = progressive disease; Pt = patient; PR = partial response; RT = radiotherapy; SC = subcutaneous; SD = stable disease; STR = subtotal resection; TCZ = tocilizumab; QD = daily; VMAT = volumetric modulated arc therapy; and VPS = ventriculoperitoneal shunt.

**Table 2:** Summary of the studies included in the systematic literature review reporting on targeted therapies for papillary craniopharyngiomas.

Author, Year	Age (Years), Sex	Prior Treatment	Systemic Targeted Treatment		Next Treatment	Outcomes	Adverse Events
			Agents	Duration (Months)			
Brastianos et al. [16] 2015	39, M	Multiple surgery STR (MTA × 4, EEA × 1)	Dabrafenib (150 mg, PO BID); trametinib (2 mg, PO QD) after 21 days	1,7 (52 days)	Surgery: STR (EEA) for consolidation tumor. resection on treatment day 38; RT (50.4 Gy in 28 fractions).	SD (after 18 months).	Low-grade fever (1 day).
Aylwin et al. [17] 2016	57, F	Surgery: STR(EEA); STR(EEA)-RT; STR(EEA)	Vemurafenib 960 mg BID	10 (3 months interruption after 3 months)	Antimicrobial therapy, surgical repair for CSF leak; re-started vemurafenib after a recurrence of the disease (6 weeks after treatment interruption)	PD (7 months after treatment initiation).	CSF leak, pneumocephalus, meningitis (due to tumor shrinkage).
Roque et al. [18] 2017	47, F	Surgery: PRc (MTA × 1); Ommaya catheter for cyst aspiration; RT (54 Gy in 30 fractions)	Dabrafenib (150 mg PO BID); trametinib (2 mg PO QD)	7	N/A	NCR (MRI—near disappearance of the tumor 7 months after treatment).	Initial intermittent fever.
Rostami et al. [19] 2017	65, M	Surgery: STR (EEA × 1)	Dabrafenib (150 mg BID); trametinib (2 mg QD) after 21 days	3,5 (15 weeks)	RT	NCR (MRI—91% reduction), clinical improvement.	Drug-induced pyrexia needs treatment interruption.
Himes et al. [20] 2018	52, M	Surgery: STR (MTA × 1); RT (36 Gy in 12 fractions)	Dabrafenib (150 mg BID → 150 mg QD due to adverse effects → 225 mg QD)	12	N/A	SD (12 months off therapy)	Arthralgia
Juratli et al. [21] 2019	21, M	Surgery (biopsy)	Dabrafenib (150 mg BID), trametinib (2 mg QD)	6	N/A	PR (80–90% reduction in the solid and cystic portions within 6 months).	N/A
Rao et al. [22] 2019	35, M	Bilateral shunts for hydrocephalus STR (MTA × 1)	Dabrafenib (150 mg BID)	24	N/A	CR of the solid component (at 24 months).	N/A
Bernstein et al. [23] 2019	60, M	Surgery: STR × 4 RT	Dabrafenib (150 mg BID); trametinib (2 mg Q.H.S) after 14 days	28	N/A	CR at 28 months (100% tumor reduction at 2 months); best clinical response after 3 months.	Prominent and widespread verrucal keratoses 2 weeks after the start of dabrafenib; resolved after discontinuation of dabrafenib and the start of combination therapy.
Di Stefano et al. [24] 2020	55, F	Surgery: STR (EEA × 1)	Dabrafenib (150 mg PO BID), trametinib (2 mg PO QD)	6,8 (208 days)	After 4.3 months of treatment, the patient underwent PBRT (52.2 Gy RBE in 29 fractions); dabrafenib and trametinib were discontinued 7 days before starting PBRT.	NCR (95% tumor reduction at 12, 7 months).	Grade 1 fatigue (CTCAE v4.0), coughing, and peripheral edema requiring temporary interruption of trametinib.
Khaddour et al. [25] 2020	39, M	Surgery: STR (EEA × 1)	Dabrafenib (150 mg PO BID), trametinib (2 mg PO QD); the treatment required interruption for 4 days due to adverse effects before later resuming the previous dosage.	9	Fractionated GKRS (Icon system; Elekta) over 5 daily fractions to a total of 25 Gy prescribed to the 50% isodose line; the maximum dose to the optic apparatus was 16 Gy.	In remission for 24 months.	Mild pyrexia (grade I fever according to CTCAE v5.0.).
Gopal et al. [26] 2020	44, M	Surgery: STR (MTA × 1)	Dabrafenib, trametinib	N/A	N/A	CR (MRI showed a decrease in size).	N/A
Butt et al. [27] 2021	32, F	Surgery: STR (MTA × 1); SRS; decompressive surgery	Dabrafenib (150 mg PO BID), trametinib (2 mg PO QD)	3	N/A	SD	Grade 2 fever; grade 2 rash.
Chik et al. [28] 2021	37, M	Surgery: STRc (EEA × 4)	Vemurafenib 960 mg BID; intermittent dose reduction after 3.7 months; 1.5-month interruption after 14.7 months.	40	STRc (MTA and EEA) RT (54 Gy/30 fractions) GKRS.	PR (MRI, 55% tumor reduction at 15 months); after the interruption, a similar reduction after 0.5 m.	Arthralgia, myalgia, elevated liver enzymes, and photosensitivity.
Nussbaum et al. [29] 2022	35, M	Surgery: STR (MTA × 1)	Initial dose: dabrafenib (75 mg, BID), trametinib (2 mg QD) Later dose: dabrafenib (250 mg BID) and trametinib (2 mg QD).	27	N/A	CR (>95% decrease in size after 21 months of treatment).	Iron deficiency, anemia, elevated values on liver function tests, the etiology of which is unclear.
Calvanese et al. [2] 2022	40, M	Surgery: NCR (EEA × 1)	Dabrafenib (150 mg BID), trametinib (2 mg QD).	5	Fractionated VMAT RT (52.2 Gy/29 fractions).	NCR (90% tumor reduction 12 months after RT).	N/A
Calvanese et al. [2] 2022	69, M	Biopsy	Dabrafenib 150 mg BID, trametinib 2 mg QD.	4	RT (52 Gy/30 fractions).	NCR (90% tumor reduction).	N/A
Lin et al. [30] 2023	59, M	N/A	Dabrafenib 150 mg BID, trametinib 2 mg QD; discontinued after 10 days due to pyrexia; the patient was continued on dabrafenib.	6,8	Dabrafenib 75 mg BID.	SD (after 3 months of follow-up).	Pyrexia increased longstanding palpitations, evaluated with 4-day Holter caused by paroxysmal atrial flutter. Dabrafenib has not been clearly associated with atrial arrhythmias.
Brastianos et al. [31] 2023	33–83 (16 Pts), M (7 Pts) F (9 Pts)	Cohort A: with/without surgery Cohort B: RT with/without other treatment (except for BRAF or MEK inhibitors).	Vemurafenib (960 mg PO BID) for 28 days in combination with cobimetinib (60 mg PO QD) for 21 days.	N/A	RT (×6 Pts) RT and surgery (×1 Pt) RT and dabrafenib (×1 Pt); off-protocol vemurafenib and cobimetinib (×1 Pt); no treatment (×7 Pts).	CR/PR (average reduction of 83%) in 15 Pts Nonresponse 1 Pt PFS 87% at 12 months and 58% at 24 months OS 100% both at 12 and 24 months.	Twelve patients had grade 3 adverse effects: rash, dehydration, increase in alkaline phosphatase levels, prolongation of corrected QT. Two patients had grade 4 adverse effects: 1 Pt → asymptomatic grade 4 increase in creatine kinase level 1 Pt → grade 4 hyperglicemia.
Shah et al. [32] 2023	57, F	Surgery: STR (EEA); IMRT (21,6 Gy in 12 fractions); STR; endoscopic transsphenoidal fenestration.	IMRT (37,8 Gy) in conjunction with 1 cycle of dabrafenib and trametinib.	N/A	Decadron (4 mg BID); gradual decadron taper to relieve brain edema.	CR (no evidence of disease recurrence 48 months after treatment).	Grade 2 acute CNS toxicity including marked fatigue and headaches, which responded to steroids; grade 2 excoriations; grade 3 vascular toxicity: diverticulitis complicated with pelvic abscess and pulmonary embolus.
Wu et al. [33] 2023	63, F	STR (MTA) GKRS (every 12 months) GTR.	Dabrafenib (150 mg BID) and trametinib (2 mg QD).	3	After a tumor-free period of 24 months, the disease recurred; the patient restarted combination therapy.	NCR (near 100% reduction size with tiny stable residual tumor) 3 months after treatment.	N/A
Wu et al. [33] 2023	75, M	STR (EEA)	Dabrafenib (150 mg BID) and trametinib (2 mg QD); the therapy was interrupted due to adverse effects.	2	N/A	PR (40% after 3 months).	Hyperglycemia and lower limb edema.

Abbreviations: BID = twice daily; CR = complete response (>95%); CSF = cerebrospinal fluid; CTCAE = common terminology criteria for adverse events; EEA = endoscopic endonasal approach; GKRS = gamma knife radiosurgery; GTR = gross total resection; IMRT = intensity-modulated radiation therapy; MTA = microscopical transcranial approach; N/A = not applicable; NCR = near-complete response (85–95%); OS = overall survival; PBRT= proton beam radiotherapy; PD = progressive disease; PFS = progression-free survival; PO = per os; PRc = partial resection; PR = partial response (<85%); Pt = patient; RT = radiotherapy; RBE = relative biological effectiveness; RS = radiosurgery; SD = stable disease; SRS = stereotactic radiosurgery; STR = subtotal resection; QD = every day; Q.H.S = nightly; and VMAT = volumetric modulated arc therapy.

## Data Availability

Data is contained within the article.
